# Vacancies and Appointments in London

**Published:** 1859-09

**Authors:** 


VACANCIES AND APPOINTMENTS IN LONDON.—Dr. Carpenter is about to resign the Professorship of Medical Jurisprudence in University College, and the chair will be filled by Dr. Harley.

The death of Dr. Samuel Griffith has rendered vacant the post of Physician-Accoucher, and Lecturer on Clinical Midwifery, to St. Thomas's Hospital.

The Chairs of Human Anatomy and Surgery, and Comparative Anatomy and Physiology, in the Royal College of Surgeons, are rendered vacant by the resignation of Professors Hewett and Busk.

